# Impact of Financial Liberalization on Banking Sectors Performance from Central and Eastern European Countries

**DOI:** 10.1371/journal.pone.0059686

**Published:** 2013-03-21

**Authors:** Alin Marius Andries, Bogdan Capraru

**Affiliations:** Faculty of Economics and Business Administration, Alexandru Ioan Cuza University of Iasi, Romania; Cinvestav-Merida, Mexico

## Abstract

In this paper we analyse the impact of financial liberalization and reforms on the banking performance in 17 countries from CEE for the period 2004–2008 using a two-stage empirical model that involves estimating bank performance in the first stage and assessing its determinants in the second one. From our analysis it results that banks from CEE countries with higher level of liberalization and openness are able to increase cost efficiency and eventually to offer cheaper services to clients. Banks from non-member EU countries are less cost efficient but experienced much higher total productivity growth level, and large sized banks are much more cost efficient than medium and small banks, while small sized banks show the highest growth in terms of productivity.

## Introduction

The opening to the outside and the internal structural reforms of the financial sector are two interdependent processes, both having as a purpose the development of a financially competitive and efficient system in order to facilitate economic growth and financial system stability.

In the present days, in the context of recent turmoil on the financial markets, there is a dispute regarding the benefits of financial liberalization. There are opinions that the financial deregulation and the increasing of the process of globalization were the main causes what amplified the recent financial crisis. Many studies evaluate the direct impact of financial deregulation on banking performance, their empirical results are also rather controversial. Some authors, such as [1], [2], [3], [4], [5], show that financial deregulation has a positive impact on banking efficiency and on the productivity of banks, while other authors consider that deregulation has a negative effect on the performance of banks, determining a decrease of technical efficiency [6] or consider that financial liberalization most often leads to financial crises [7].

Combining insights from the liberalization – efficiency and financial openness – stability literatures, we develop a unified framework to assess how regulation, supervision and other institutional factors may affect the performance of banking systems in 17 countries from Central and Eastern Europe for the period 2004–2008. This study seeks to address two key questions. What variables influence the performance of banks from Central and Eastern European countries? Did the financial liberalization and reforms in the banking system have a notable influence on bank performance?

Actually, we analyze the impact of financial liberalization and reforms in the banking system as well as the associated changes in the industry structure on the banking performance, measured in terms of cost efficiency and total productivity growth index. To do this, we develop a two-stage empirical model that involves estimating banks’ performance in the first stage and assessing its determinants in the second one.

The importance and originality of this paper consist in assessing the CEE banking systems in a period when there were two waves of EU enlargements and the first influences of the recent international financial crises had appeared. Our sample of countries could be split into three categories: EU members, EU candidates and other potential EU candidates. The results of our papers are important in the context of the present financial turmoil; therefore, in the end of the paper, we try to develop some policy recommendations for both policy makers from CEE countries and EU ones. The evidence of our research could also be useful for banks’ strategies of internationalization.

Cross-country efficiency studies in the banking industry have attracted a lot of attention. For banks, efficiency implies improved profitability, greater amount of funds channeled in, better prices and services quality for consumers and greater safety in terms of improved capital buffer in absorbing risk [8].

Studies of the impact of deregulation upon efficiency have found different results. Evidences from Taiwan [9], Korea [10], Norway [1], Turkey [11] and Thailand [12] proved improvements in efficiency, while in the case of Spain [13] and US [14], [15] found that deregulation have a negative impact upon efficiency.

Studies focused on the case of developing countries from Central and Eastern European countries explore various issues including the impact of ownership and privatization [16], [17], competition [5], [18] and the bank reforms and regulation [19], [20] on the banks’ efficiency. Cross-country efficiency studies have also become more common for CEE banking systems as the success of the economic transition in the 1990s, the progress of privatization and similar development paths fostered by the EU accession process have boosted the interest of researchers in the region [21].

The creation of an effective and solid financial system constituted an important objective of the process of reform and transition from a centralized economy to a market economy in CEE countries. The liberalization of prices, the liberalization of the circulation of goods, services and capital, the deregulation of financial systems, globalization and the mutations on the level of the economic, social and political environment had a significant impact on the development of the CEE banking system [22]. The banking systems in the developing countries suffered ample mutations with the purpose of creating some efficient banking institutions, with a high degree of soundness capable of facilitating economic growth.

Most studies focused on the banking system in Central and Eastern Europe (CEE) are only performed at the level of one state and do not offer comparative information regarding the efficiency and productivity growth of banks in these states. However, in recent years, several papers have published comparative analyses highlighting the impact of banking system reform, the evolution of banking structure, competition and privatization on banks’ efficiency (see e.g. [5], [16], [17], [18], [23], [24], [25], [26], [27], [28], [29],).

Fang et al. find that the institutional development, proxied by progress in banking regulatory reforms, privatization and enterprise corporate governance restructuring, has a positive impact on bank efficiency [30].

Brissimis et al. examine the relationship between banking system reform and bank performance – measured in terms of efficiency, total factor productivity growth and net interest margin – accounting for the effects through competition and bank risk-taking [5]. The model is applied to bank panel data from ten newly acceded EU countries. The results indicate that both banking system reform and competition exert a positive impact on the bank efficiency, while the effect of reform on total factor productivity growth is significant only by the end of the reform process.

Pasiouras et al. uses stochastic frontier analysis to provide evidence on the impact of regulatory and supervision framework on bank efficiency based on a dataset consisting of 2853 observations from 615 publicly quoted commercial banks operating in 74 countries during the period 2000–2004 [31]. Their results suggest that banking regulations that enhance market discipline and empower the supervisory power of the authorities increase both cost and profit efficiency of banks. In contrast, stricter capital requirements improve cost efficiency but reduce profit efficiency, while restrictions on bank activities have the opposite effect, reducing cost efficiency but improving profit efficiency.

The rest of the paper is organized as follows: in section 2 we explain the methodology used to measure the impact of financial liberalization on the bank efficiency and productivity growth and we discuss the data and the variable selection. Thereafter, the results of the empirical analysis are presented and discussed in section 3. The main conclusions are drawn in section 4.

## Methodology and Data

In this section we discuss the empirical model used to investigate the impact of financial liberalization on bank performance. Then we explain our measures of bank performance: cost efficiency and productivity growth. The discussion of data and control variables follows afterwards.

### 2.1. Estimable Model

The purpose of the estimable model outlined in this section is to capture the effects of financial liberalization, reforms in the banking system and the associated changes in the industry on bank performance. We also include a range of bank-specific variables that have been used in previous empirical studies that examine the drivers of bank performance. The model is specified as:
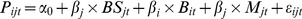
(1)where the subscripts i, j, t denote bank i, country j, and year t; 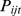 – performance indicators of the banks; 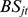 – banking system specific variables; 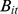 – bank-specific variables; 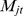 – macroeconomic variables; 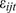 – error term.

#### 2.1.1. Measures of banks performance

Bank performance is proxied alternatively by cost efficiency (EFF) and total productivity growth index (TFPCH). These indicators have been used widely in previous empirical literature concerned with the measurement and determinants of the bank performance in developing countries [5], [17], [18], [32]. The analysis of the efficiency and productivity of banks can be performed both by means of parametrical methods and of non-parametrical methods. For a comparison of these methods see [2], [33], [34].

In line with [35] we measure cost efficiency as how close a bank’s cost is to what best practice banks cost would be for producing the same output bundle under the same conditions. As costs functions are not directly observable, inefficiencies are measured relative to an efficient cost frontier. When assessing the impact of financial liberalization on banking performance we also use the total productivity growth index what measures the modification of total productivity of the factors between the two periods of time, by calculating the ratio between the distances from each point observed in the respective technology.

In the estimation of the cost efficiency level of the banks in CEE countries we used the SFA Method and applied the model developed by [36]. The cost frontier can be expressed thus:
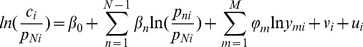
(2)where: *y_it_*– outputs vector; 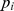 – prices of inputs vector; 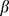 and 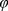 – independent variable coefficients; 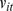 – random error 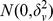; 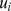 – error variable that follows a normal-truncated distribution; *t –* time component.

The cost frontier indicates the minimum cost, 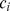, which a decisional unit can register in order to produce a quantity of outputs, 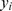, considering the prices of inputs, 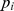. The cost efficiency level is given by the ratio between the minimum cost and the cost registered by the decisional unit and it is calculated as:
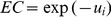
(3)


The SFA method assumes that the inefficiency component of the error term is positive and thus the high costs are associated with a high level of inefficiency.

In the order to quantify the total productivity growth we estimated the Malmquist index with the help of the DEA-type linear programming method, a method that was introduced by [37] and developed by [38]. The Malmquist index measures the modification of total productivity of the factors between two periods of time, by calculating the ratio between the distances from each point observed in the respective technology.

Färe et al. proposed in [37] the following form for the Malmquist index (output oriented), between two periods of time t (basic period) and (t+1) (current period):
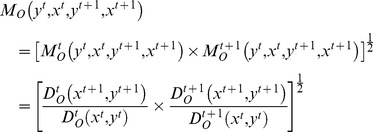
(4)where 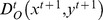 represents the distance from the point observed in the period t+1 to the frontier of the technology of period t. 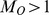 indicate an increase of the total productivity of factors from one period to another, while 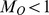 corresponds to a decline of total productivity of factors.

In the empirical analysis of the mutations on the level of the productivity of banks we have to calculate four distance measures that occur in equation (4) for each pair of adjacent periods of time. Having at disposal the panel sets of data, we can calculate the distance functions with the help of the DEA method. For the bank “i”, i = 1, 2, …, N the DEA linear programming problems, under the assumption that the technologies have constant returns to scale, can be written:
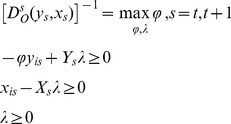
(5)

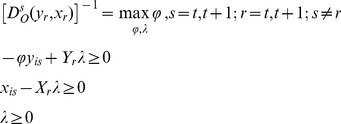
(6)


The linear programming problems must be solved N times, once for each company in the ensemble. The introduction of solutions to the problems in relation (4) allows for the estimation of the Malmquist index of productivity.

#### 2.1.2. Banking system characteristics

Because the purpose of this analysis is to analyze the connection between the performance of banks and the degree of financial liberalization of the banking system, the first set of banking system characteristics considered in the model includes the following variables: Banking reform and interest rate liberalization indicator (BREF), Financial Openness Index (KOPEN), Asset share of state-owned banks (ASSB) and Asset share of foreign-owned banks (ASFB).

The Banking reform and interest rate liberalization indicator is compiled by the EBRD with the primary purpose of assessing the progress of the banking systems of formerly communist countries and quantifies and qualifies the degree of liberalization of the banking industry [5]. This indicator provides a ranking of progress in liberalization and institutional reform of the banking system, on a scale of 1 indicating little progress in reform to 4 representing a level that approximates the institutional standards and norms of an industrialized market economy [18].

In order to assess the level of financial openness we use the Chinn-Ito index that measures the country’s degree of capital account openness. The index is based on the binary dummy variables that codify the tabulation of restrictions on cross-border financial transactions reported in the IMF’s Annual Report on Exchange Arrangements and Exchange Restrictions [39].

Following previous studies that focus on banks’ performance [27], [40], [41], we control for cross-country differences in the national structure and competitive conditions of the banking system, using the following measures: i) Asset share of state-owned banks (ASSB) that are quantified as percentage of asset share of state-owned banks in total assets of banking system, the state includes the federal, regional and municipal levels, as well as the state property fund and the state pension fund (state-owned banks are defined as banks with state ownership exceeding 50 per cent, end-of-year); ii) Asset share of foreign-owned banks (ASFB) that show the share of banks with foreign ownership exceeding 50 per cent in total bank system assets. We use these indicators to assess the impact of state and foreign ownership on performance differences in national banking systems; iii) Number of banks (NB); iv) The percentage share of the three largest banks (CR3), ranked according to assets, in the sum of the assets of all the banks in that banking system; v) Herfindahl-Hirschmann index (HHI) that is calculated as the sum of the squares of all the banks’ market shares in terms of total assets.

We measure bank stability using Z-score, which is a very popular indicator in recent literature concerned with the measurement and determinants of soundness and safety of banks [42]. The Z-score is calculated as:
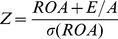
(7)


ROA is the bank’s return on assets, E/A represents the equity to total assets ratio and 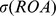 is the standard deviation of return on assets. A higher Z-score implies a lower probability of insolvency, providing a direct measure of soundness that is superior to analyzing leverage.

The data used to quantify these indicators have been taken from EBRD and ECB reports.

#### 2.1.3. Bank-specific variables

The economic literature pays a great deal of attention to the performance of banks, expressed in terms of efficiency, productivity, competition, concentration, soundness and profitability.

The use of risk indicators in the analysis of bank performance has gained in the past decades a special attention because the control on banks’ risks is one of the most important factors the profitability of the bank depends on [43].

Following the empirical literature, we use the Return on Assets (ROA) to reflect the bank’s management ability to use the resources the bank disposes of for the purpose of optimizing profit. Bank capital adequacy is measured as the equity to assets ratio, quantified as the value of total equity divided by the value of total assets.

To express the risk profile of the banks we use two different types of risk: credit risk measured as ratio of loan-loss provisions to total loans (LLR_GL) and liquidity risk measured as ratio of liquid assets to total deposits and borrowing funds (LA_TD). Another variable used in the analysis is the bank’s size measured as logarithm of total assets (TAL).

The data used in the analysis are taken from the annual reports of the banks and from the Fitch IBCA’s BankScope database.

#### 2.1.4. Macroeconomic variables

In line with the previous literature [31], [44], [45], [46], we include a variety of macroeconomic variables in our model. The macroeconomic variables used in our analysis are: GDP growth rate – Growth in real GDP in per cent (GDP_G), Inflation rate - change in annual average retail/consumer price level in per cent (IR), Level of financial intermediation – domestic credit provided by banking system percentage of GDP (FIN_INT), and Interest rate spread – lending rate minus deposit rate percentage (IRS).

In order to quantify the effects of structural reforms, we also use two governance indicators developed by Kaufmann et al. to proxy institutional differences: rule of law (ROL) and regulatory quality (RQ) [47]. Rule of law is an indicator of the extent to which agents have confidence in and abide by the rules of society while regulatory quality is an indicator of the ability of the government to formulate and implement sound policies. These indicators are assessed on a scale of about −2.5 to 2.5 with higher values corresponding to a ‘better’ regulatory environment.

Improvements in the regulatory quality help banks if it is accompanied by more adequate banking supervision. The quality of the rule of law affects cost efficiency through the effectiveness and predictability of the judiciary. There is a growing literature that points to the importance of institutions for an efficient operation of the financial system. This literature argues that better institutions positively affect bank efficiency (see also [48]). The data used to quantify this indicator have been taken from EBRD, World Bank and ECB reports.

### 2.2. Data

This study seeks to undertake this assessment by examining banking efficiency and productivity growth in 17 countries from Central and Eastern Europe (Albania, Bosnia and Herzegovina, Bulgaria, Croatia, Czech Republic, Estonia, Hungary, Latvia, Lithuania, Macedonia, Republic of Moldova, Montenegro, Poland, Romania, Serbia, Slovakia and Slovenia). We omit Belarus and Ukraine from our study because we could not obtain sufficient data. All bank-level data used are obtained from the BankScope database and are reported in Euros. To be included in our sample, a bank has to have a minimum of 3 years of continuous data to obtain reliable efficiency estimates [27]. The selection process yields an unbalanced panel with 236 banks (730 observations) for the 2004–2008 period.

In the literature in the field there is no consensus regarding the inputs and outputs that must be used in the analysis of the efficiency and productivity growth of commercial banks [2]. In our paper, bank inputs and outputs are defined according to the value-added approach, originally proposed by Berger and Humphrey [49], which suggests using deposits as outputs since they imply the creation of value added. Following [44], [45], we used the following set of inputs and outputs in order to quantify the efficiency and mutations on the level of the productivity of banks: Loans, Other earning assets and Demand deposits – as outputs; Personnel expenses, Fixed assets and Financial capital (sum of total deposits, total money market funding, total other funding and equity) – as inputs. Input prices are obtained as Total personnel expenses over Total assets, Other operating expenses over Fixed assets and Interest expenses over Financial capital.

Table 1 and 2 present the mean values for the banking system characteristics, bank-specific variables and macroeconomic variables.

**Table 1:** Means of banking system characteristics, bank-specific variables and macroeconomic variables by year.

YEAR	BREF	KOPEN	ASSB	ASFB	CR3	NB		HHI			Z_SCORE	
**2004**	3.31	1.05	8.52	66.29	0.62	32.61		1136.37			8.76	
**2005**	3.42	1.14	8.24	72.42	0.61	33.60		1097.16			9.63	
**2006**	3.43	1.36	7.04	77.43	0.59	32.52		1142.94			9.38	
**2007**	3.49	1.43	6.45	77.18	0.65	32.59		1122.89			9.71	
**2008**	3.56	1.49	7.33	80.07	0.72	33.23		1088.31			10.59	
**Average**	3.44	1.30	7.51	74.68	0.64	32.91		1116.78			9.61	
**YEAR**	**ROA**	**LA_TD**	**LLR_GL**	**TA**	**GDP_G**	**IR**	**FIN_INT**		**IRS**	**ROL**		**RQ**
**2004**	1.44	42.00	5.95	1583.52	6.08	5.88	33.52		6.24	0.21		0.50
**2005**	1.77	39.70	5.46	1938.34	5.57	5.39	39.82		5.96	0.15		0.48
**2006**	1.48	36.84	4.35	2374.87	6.54	5.26	46.35		5.00	0.17		0.51
**2007**	1.31	34.20	3.48	2948.24	6.39	5.21	54.35		4.53	0.21		0.55
**2008**	0.71	28.52	4.00	3233.63	3.92	8.36	59.02		4.24	0.25		0.60
**Average**	1.34	36.24	4.63	2415.72	5.70	6.03	46.62		5.19	0.20		0.53

Notes: BREF = Banking reform and interest rate liberalization indicator; KOPEN = Financial Openness Index; ASSB = Asset share of state-owned banks; ASFB = Asset share of foreign-owned banks; CR3 = The percentage share of the three largest banks; NB = Numbers of banks; HHI = Herfindahl - Hirschmann index; Z_SCORE = Z-Score index; ROA = Return on Assets; LLR_GL = Ratio of loan-loss provisions to total loans; LA_TD = Ratio of liquid assets to total deposits and borrowing funds; TA = Total assets; GDP_G = GDP growth rate; IR = Inflation rate; FIN_INT = Level of financial intermediation; IRS = Interest rate spread; ROL = Rule of law; RQ = Regulatory quality.

**Table 2:** Means of banking system characteristics, bank-specific variables and macroeconomic variables by country.

COUNTRY_CODE	KOPEN	BREF	ASSB	ASFB			CR3		NB		HHI			Z_SCORE	
**ALBANIA**	−0.72	2.76	2.88	92.78			0.82		16.40		192.00			9.58	
**BOSNIA-HERZEGOVINA**	1.50	2.76	2.72	90.92			0.59		32.00		919.00			13.13	
**BULGARIA**	1.26	3.70	1.98	80.48			0.51		32.00		758.60			7.28	
**CROATIA**	1.17	4.00	3.96	90.92			0.61		36.20		1322.20			14.36	
**CZECH REPUBLIC**	2.50	3.94	2.58	84.70			0.64		36.40		1095.20			10.33	
**ESTONIA**	2.50	4.00	0.00	98.70			0.95		13.60		3609.80			9.49	
**HUNGARY**	2.45	4.00	5.64	75.34			0.71		41.00		814.80			6.05	
**LATVIA**	2.50	3.82	7.28	59.86			0.55		24.40		1166.20			8.04	
**LITHUANIA**	2.45	3.62	0.00	91.62			0.79		13.20		1829.20			11.46	
**MACEDONIA (FYROM)**	0.12	2.76	1.54	66.16			0.76		19.20		1618.20			13.40	
**MOLDOVA REP. OF**	−1.14	2.82	14.16	26.50			0.55		15.80		1167.20			10.04	
**MONTENEGRO**	0.12	2.60	4.30	74.78			–		10.40		–			–	
**POLAND**	0.12	3.62	20.42	74.36			0.60		63.00		628.60			7.05	
**ROMANIA**	2.18	3.12	6.24	76.12			0.66		31.80		1070.80			3.74	
**SERBIA**	0.12	2.68	18.78	66.64			–		37.80		650.00			–	
**SLOVAKIA**	1.13	3.70	1.06	97.84			0.77		24.00		1128.00			11.50	
**SLOVENIA**	2.13	3.30	13.38	26.38			0.59		24.60		1334.20			14.49	
**Average**	1.30	3.44	7.51	74.68			0.64		32.91		1116.78			9.61	
**COUNTRY_CODE**	**ROA**	**LA_TD**	**LLR_GL**		**TA**	**GDP_G**		**IR**		**FIN_INT**		**IRS**	**ROL**		**RQ**
**ALBANIA**	0.84	50.61	2.22		304.26	5.78		2.66		21.96		7.10	−0.74		−0.05
**BOSNIA-HERZEGOVINA**	0.95	52.10	5.53		267.14	5.91		5.02		44.04		4.81	−0.51		−0.28
**BULGARIA**	1.77	37.12	3.17		918.67	6.36		7.88		53.30		5.97	−0.09		0.65
**CROATIA**	1.00	36.15	6.43		1448.90	4.21		3.50		61.48		8.36	0.11		0.47
**CZECH REPUBLIC**	1.00	31.95	2.90		5851.63	5.24		3.30		40.90		4.57	0.86		1.09
**ESTONIA**	1.58	54.35	1.57		4580.57	6.32		5.71		70.56		2.68	1.05		1.37
**HUNGARY**	1.37	28.19	1.97		4404.06	2.84		5.64		55.04		2.06	0.89		1.20
**LATVIA**	1.31	39.16	1.56		1242.22	7.39		8.99		76.60		4.33	0.69		1.00
**LITHUANIA**	1.22	26.25	1.51		2411.93	7.12		4.85		47.96		2.35	0.65		1.08
**MACEDONIA (FYROM)**	2.05	40.91	6.81		249.92	4.57		3.04		31.62		5.72	−0.36		0.05
**MOLDOVA REP. OF**	3.46	35.45	4.38		101.36	6.10		12.48		29.12		5.01	−0.48		−0.35
**MONTENEGRO**	1.31	36.66	2.85		226.94	6.96				49.42		5.21	−0.34		−0.36
**POLAND**	1.59	26.91	6.29		5975.97	5.40		2.71		40.50		3.54	0.47		0.79
**ROMANIA**	1.04	41.33	2.08		2123.97	7.18		8.03		27.18		9.72	−0.06		0.38
**SERBIA**	1.00	47.90	11.47		478.34	6.30		11.63		32.26		–	−0.61		−0.38
**SLOVAKIA**	1.03	34.27	3.84		3341.37	7.39		4.42		38.24		4.36	0.56		1.12
**SLOVENIA**	1.08	20.43	5.08		3120.77	4.98		3.56		66.92		3.80	0.93		0.80
**Average**	1.34	36.24	4.63		2415.72	5.70		6.03		46.62		5.19	0.20		0.53

Notes: BREF = Banking reform and interest rate liberalization indicator; KOPEN = Financial Openness Index; ASSB = Asset share of state-owned banks; ASFB = Asset share of foreign-owned banks; CR3 = The percentage share of the three largest banks; NB = Numbers of banks; HHI = Herfindahl-Hirschmann index; Z_SCORE = Z-Score index; ROA = Return on Assets; LLR_GL = Ratio of loan-loss provisions to total loans; LA_TD = Ratio of liquid assets to total deposits and borrowing funds; TA = Total assets; GDP_G = GDP growth rate; IR = Inflation rate; FIN_INT = Level of financial intermediation; IRS = Interest rate spread; ROL = Rule of law; RQ = Regulatory quality.

When analyzing the means of determinants of efficiency value we can observe that the degree of financial liberalization of the banking system has continuously increased during the assessed period. Thus the level of the banking reform and interest rate liberalization indicator (BREF), Financial Openness Index (KOPEN) and asset share of foreign-owned banks (ASFB) increased and the level of asset share of state-owned banks (ASSB) due to the privatization process and the increase of foreign capital (the last two determinants are correlated). The number of banks was relatively stable, the concentration ratio of the first 3 banks continuously grew, but the evolutions of HHI denote a moderate competition towards high competition, being relatively stable. The stability of the entire banking systems, from the perspective of insolvency probability, has increased continuously as Z-score relieves. The explanations could be the process of harmonization with the EU acquis, which implies a better banking regulation framework. We consider that the evolutions of these determinants were influenced by the process of European integrations, because some of the countries assessed are EU members, some of them are EU candidates and others potential EU candidates.

The bank-specific variables had different evolutions. Thus we can observe a decrease of ROA in the context of an ample growth of total bank assets. The risk profile of the banks evaluated as following: the ratio of loan-loss provisions to total loans (LLR_GL) and liquidity risk measured as ratio of liquid assets to total deposits and borrowing funds (LA_TD) have decreased, indicating a loss in bank liquidity, but a better credit risk situation.

### 2.3. Estimation Approach

The empirical models used in the specialty literature use a two-stage procedure: in the first stage the level of cost efficiency and total productivity growth is estimated and in the second stage the regression analysis is applied in which the levels of cost efficiency and total productivity index are dependent variables.

The empirical model specified in the equation is estimated using the panel least square fixed effects methodology. We use the fixed effects model, since we focus on a limited number of countries, for which we want to assess country-specific differences with respect to the relationship between financial liberalization and bank performance. For this purpose, performance scores are regressed on a set of common explanatory variables; a positive coefficient implies efficiency increase whereas a negative coefficient means an association with an efficiency decreases. The empirical model is tested for each of the two measures of banking performance, i.e. cost efficiency and total productivity growth.

The research strategy follows the specific-to-general approach. We start by investigating the relationship among cost efficiency and Banking reform and interest rate liberalization indicator (BREF) and Financial Openness Index (KOPEN). Next, we include all other banking system characteristics, bank-specific variables and macroeconomic variables one by one to test the stability of the main independent variables BREF and KOPEN. A second set of models is estimated using total productivity growth index as dependent variable.

## Empirical Results

### 3.1. Efficiency and Productivity Level

Table 3 presents the estimates of the cost efficiency level and total productivity growth index, showing the results by country and year.

**Table 3:** Means of cost efficiency level and total productivity growth index by country and year.

COUNTRY	EFF	TFPCH
**ALBANIA**	.9330446	.9047500
**BOSNIA-HERZEGOVINA**	.8480512	1.9773438
**BULGARIA**	.9111905	1.0845972
**CROATIA**	.9439455	1.3550217
**CZECH REPUBLIC**	.9688820	1.0250263
**ESTONIA**	.8788302	1.3021500
**HUNGARY**	.9439129	1.2320263
**LATVIA**	.8948106	1.3645313
**LITHUANIA**	.8861573	1.2571071
**MACEDONIA (FYROM)**	.8584147	1.1361250
**MOLDOVA REP. OF**	.9020564	1.5848250
**MONTENEGRO**	.8389457	1.9448500
**POLAND**	.9385431	1.0464130
**ROMANIA**	.8426061	1.2679079
**SERBIA**	.8300287	1.7130833
**SLOVAKIA**	.8691180	1.1644500
**SLOVENIA**	.8920361	1.2795000
**Average**	.8983056	1.3252415
**YEAR**	**EFF**	**TFPCH**
**2004**	.8866425	
**2005**	.8924896	1.3511102
**2006**	.8983264	1.3626441
**2007**	.9041426	1.2441144
**2008**	.9099270	1.3430975
**Average**	.8983056	1.3252415

Notes: EFF = Cost efficiency; TFPCH = Total productivity growth index.

From empirical results we see that the average cost efficiency of banks in Central and Eastern European countries grew in the period analyzed, from an average value of 0.8866 in 2004 to 0.9099 in 2008, but there is significant variation across the banking systems of the Central and Eastern European countries in terms of cost efficiency level. Similar to [30], our results show that the highest level of efficiency is recorded in the banking systems from the Czech Republic and the lowest is recorded in Serbia. The higher increase of total productivity growth index during 2004–2008 was recorded in Bosnia and Herzegovina, Montenegro, Serbia and Republic of Moldova. Only Albania recorded a decrease of total productivity growth index during the analyzed period. Our results are in line with previous results obtained by [18] and [20].

Table no. 4 also shows the average cost efficiency and productivity growth results for banks of different size. Following [50] we classified banks into 3 different categories considering the size of banks: small if it has total assets <1 000 mil EUR; medium if it has total assets >1000 mil EUR and <10 000 mil EUR; and large if it has total assets >10 000 mil EUR. We also classified the banking systems in two different categories considering the status of the country: member or non-member of the European Union.

**Table 4:** Means of cost efficiency level and total productivity growth index by size of banks and status of country.

Size ofbanks	EFF	TFPCH	Status ofcountry	EFF	TFPCH
**Small**	0.8968371	1.4637117	**Non-member** **of EU**	0.8791212	1.5655112
**Medium**	0.8943662	1.1389842	**Member of EU**	0.9099207	1.1797721
**Large**	0.9339452	1.0363692	**All**	0.8983056	1.3252415

Notes: EFF = Cost efficiency; TFPCH = Total productivity growth index.

Thus the results show that, on average, banks from a non-member country are less cost efficient but experienced much higher total productivity growth level during 2004–2008 period. In non-member countries, these productivity gains could be due to technological progress, rather than to an improvement in efficiency. Large sized banks are much more cost efficient than medium and small banks, while small sized banks show the highest growth in terms of productivity. This suggests that small sized banks are able to generate strong profits possibly by operating in the high value added segments of the markets while incurring higher costs at the same time.

### 3.2. Determinants of Efficiency

Table 5 and 6 report the key empirical results of the second stage analysis based on the estimation of Panel OLS models, using cost efficiency and total productivity growth index as the dependent variables.

**Table 5:** Determinants of cost efficiency.

Dependend variable: Cost efficiency				
Model	1	2	3	4
BREF	0.039741*** (0.001787)	0.030006*** (0.002636)	0.015697*** (0.003028)	0.00542** (0.002374)
KOPEN	0.007113*** (0.000522)	0.005852*** (0.000558)	0.003952*** (0.000598)	0.001734*** (0.000516)
ASSB		2.38E−07 (0.000115)	0.000238* (0.000124)	−0.000353** (0.000146)
ASFB		0.000337*** (3.60E−05)	0.00019*** (4.66E−05)	1.74E−06 (5.11E−05)
NB		−8.01E−05 (0.000117)	3.45E−05 (0.000129)	0.000173 (0.000111)
CR3		0.013322*** (0.0037)	0.002114 (0.003993)	−0.020292*** (0.003629)
HHI		−0.0000141*** (3.18E−06)	−0.0000141*** (3.33E−06)	0.00000846*** (2.90E−06)
Z_SCORE		0.000274*** (9.80E−05)	0.000253** (0.000102)	−5.40E−05 (8.34E−05)
ROA			−0.000775** (0.000331)	−0.000518** (0.000257)
LLR_GL			−0.000276** (0.000132)	−0.000201** (0.000101)
LA_TD			−0.00012*** (2.69E−05)	−2.16E−05 (2.09E−05)
TAL			0.007326*** (0.000694)	0.000869 (0.000618)
GDP_G				−0.000379*** (0.000124)
IR				0.000786*** (0.00016)
FIN_INT				0.000501*** (3.65E−05)
IRS				−0.001449*** (0.000259)
ROL				−0.008359* (0.004266)
RQ				0.008363 (0.005092)

Note: Standard deviations are presented between brackets; *, **, ***indicates significance levels at 10%, 5% and 1%; BREF = Banking reform and interest rate liberalization indicator; KOPEN = Financial Openness Index; ASSB = Asset share of state-owned banks; ASFB = Asset share of foreign-owned banks; CR3 = The percentage share of the three largest banks; NB = Numbers of banks; HHI = Herfindahl - Hirschmann index; Z_SCORE = Z-Score index; ROA = Return on Assets; LLR_GL = Ratio of loan-loss provisions to total loans; LA_TD = Ratio of liquid assets to total deposits and borrowing funds; TA = Total assets; GDP_G = GDP growth rate; IR = Inflation rate; FIN_INT = Level of financial intermediation; IRS = Interest rate spread; ROL = Rule of law; RQ = Regulatory quality.

**Table 6:** Determinants of total productivity growth.

Dependend variable: Total productivity growth index				
Model	1	2	3	4
BREF	0.400634*** (0.024241)	0.327468*** (0.087397)	0.259045*** (2.58439)	0.395788*** (0.14925)
KOPEN	0.083508* (0.047573)	0.026943* (0.040201)	0.026183* (0.545913)	0.12306* (0.064195)
ASSB		0.014873 (0.010082)	0.019555* (1.818648)	0.016693 (0.012175)
ASFB		−0.000205 (0.002812)	0.002522 (0.791936)	0.000212 (0.004411)
NB		−0.008881* (0.005139)	−0.006295 (−1.154973)	−0.002406* (0.006392)
CR3		0.202387 (0.313091)	0.062689 (0.174837)	−0.023763* (0.374783)
HHI		6.50E−05 (0.000102)	3.45E−05 (0.324424)	0.000125 (0.000119)
Z_SCORE		0.008228 (0.009468)	0.012189* (1.226219)	0.010519* (0.012144)
ROA			0.004147 (0.135944)	−0.037154* (0.033597)
LLR_GL			−0.002466 (−0.19703)	−0.001239 (0.013331)
LA_TD			0.002543 (1.271163)	0.000947 (0.002095)
TAL			−0.027018 (−0.98238)	−0.026066 (0.028349)
GDP_G				0.03044** (0.014903)
IR				0.003246 (0.01937)
FIN_INT				−0.002651 (0.003325)
IRS				−0.035951 (0.026044)
ROL				−0.152544 (0.387756)
RQ				−0.438561 (0.455659)

Note: Standard deviations are presented between brackets; *, **, ***indicates significance levels at 10%, 5% and 1%; BREF = Banking reform and interest rate liberalization indicator; KOPEN = Financial Openness Index; ASSB = Asset share of state-owned banks; ASFB = Asset share of foreign-owned banks; CR3 = The percentage share of the three largest banks; NB = Numbers of banks; HHI = Herfindahl - Hirschmann index; Z_SCORE = Z-Score index; ROA = Return on Assets; LLR_GL = Ratio of loan-loss provisions to total loans; LA_TD = Ratio of liquid assets to total deposits and borrowing funds; TA = Total assets; GDP_G = GDP growth rate; IR = Inflation rate; FIN_INT = Level of financial intermediation; IRS = Interest rate spread; ROL = Rule of law; RQ = Regulatory quality.

As for the effect of banking system characteristics, we found that a higher level of the Banking reform and interest rate liberalization indicator (BREF) and Financial Openness Index (KOPEN) improves cost efficiency, suggesting that banks in countries with higher level of liberalization and openness are able to increase cost efficiency and finally to offer cheaper services to clients. Our results are in line with [5] for new accepted EU countries, with [27] for transition economies and [31] for 74 countries, but contrary like those of [18], [46] for some CEE countries. Like [27], our results show that a higher share of state-owned banks (ASSB) has a negative impact on the level of banks’ cost efficiency. The level of Asset share of foreign-owned banks (ASFB) has no statistically significant impact on the level of banks’ cost efficiency. This result contradicts those of [27] that demonstrated that privatised banks with majority foreign ownership are the most efficient and those with domestic ownership are the least and [26] that show that banks with higher foreign bank ownership involvement were associeted with lower inefficiency.

The results show that the level of Banking reform and interest rate liberalization indicator (BREF) and Financial Openness Index (KOPEN) have a positive impact on the total productivity growth. The Z-score is positively correlated with total productivity, demonstrating that the total productivity depends on the soundness and safety of banks.

With regard to the impact of structure of banking systems, results show that higher concentration quantified by means of the Herfindahl-Hirschmann index (HHI) improves cost efficiency, while the percentage share of the three largest banks (CR3) has a negative impact on the cost efficiency level. The mean value for these two indicators during the period assessed does not prove significant changes in the banking structure and level of competition. This evidence could suggest that the competition was not one of the most important factors of improving cost efficiency, being in contradiction with the traditional view and previous results [51].

As regards the impact of bank-specific variables, the results show that the level of Return on Assets (ROA) has a statistically significant and negative impact on both cost efficiency and total productivity growth. The level of credit risk measured as the ratio of loan-loss provisions to total loans (LLR_GL) negatively influences cost efficiency.

Turning to the effect of macroeconomic variables, we observe that GDP growth rate had a negative impact on cost efficiency, maybe because under expansive demand conditions, managers are less focused on the expenditure control and therefore become less cost efficient. Another explanation could be that the increase in credit markets involves higher capital cost, an increase in operating expenses and cost with fixed assets. This results are in line with [52], [53].

From another point of view, the decrease of GDP growth rate improves the total productivity of banks. This could be a reason for foreign-owned banks to maintain their exposure on these markets in case of economic decrease, but with the condition of maintaining the soundness and safety of banks. We also found a negative and significant relationship among Inflation rate (IR), Interest rate spread (IRS) and level of Rule of law (ROL) and bank cost efficiency.

Our results show that the level of Financial intermediation has a positive effect on the bank performance, meaning that a low level of financial intermediation hampers banking performance.

### Conclusions

From our analysis it results that the Financial liberalization improves cost efficiency of banks from Central and Eastern European countries with higher level of liberalization and openness are able to increase cost efficiency and finally to offer cheaper services to clients. These facts are in compliance with the Single European Market principles and demonstrate that EU new member states, candidate states and potential candidate states banking market mechanisms could achieve their objective of lowering and harmonization of banking services prices. In this case, from a banking policy perspective, we consider that the EU enlargement could continue in Central and Eastern European countries and could add benefits for the EU banking market.

In exchange, the level of Asset share of foreign-owned banks has no statistically significant impact on the level of bank cost efficiency. This could mean that the dominance of foreign banks on the market does not increase cost efficiency, but the best practices that they brought in the banking systems. From the policy perspectives, these results suggest that, in the case of new member countries, foreign-owned banks have no influence on increasing cost efficiency by means of their own activity and dominance on the market, but perhaps by means of their best practices that domestic banks must adopt for competing them.

In what concerns the effect of financial reform on the total productivity growth of banks from CEE countries, the results show that the level of Banking reform and interest rate liberalization indicator has a positive impact on the total productivity growth. Also, the results suggest that the important factors shaping the total productivity are merely the banking system characteristics and bank-specific variables, and the only macroeconomic variable with impact is the GDP growth rate.

Overall, in order to promote efficiency and productivity, monetary authorities from CEE countries should enhance their efforts to continue the reform of the financial services regulatory and supervisory framework. At the same time, banking markets should remain open, encouraging the entry of foreign banks for improving best practices and for increasing the benefit from technological spillovers brought by them. For a sustainable improvement of cost efficiency and total productivity of banks, the focus should be on the improvements of managerial practices, especially in domestic small and medium banks. Policy makers should also be concerned about improving the liquidity level.

Furthermore, our results indicate that policy makers in EU could take into account the follow-up of the process of enlargement in some countries from CEE, because their banking markets have a good potential in adapting the Single European Markets principles. Foreign banks could maintain their exposures or enter the CEE markets because there is a good perspective for total productivity growth and the stability of the banking systems has increased.
